# Different controllers for suppressing oscillations of a hybrid oscillator via non-perturbative analysis

**DOI:** 10.1038/s41598-023-50750-9

**Published:** 2024-01-03

**Authors:** Galal M. Moatimid, A. T. El-Sayed, Hala F. Salman

**Affiliations:** 1 Department of Mathematics, Faculty of Education, Ain Shams University, Cairo, Egypt; 2 Department of Basic Science, Modern Academy for Engineering and Technology, Elmokattam, Egypt; 3 Department of Basic Sciences, Faculty of Computers and Artificial Intelligence, Cairo University, Giza, Egypt

**Keywords:** Engineering, Mathematics and computing

## Abstract

To arrive at an equivalent linear differential equation, the non-perturbative approach (NPA) is established. The corresponding linear equation is employed for performing the structural analysis. A numerical computation demonstrates a high consistency with the precise frequency. The correlation with the numerical solution explains the reasonableness of the obtained solutions. For additional nonlinear kinds of oscillation, the methodology gives an exact simulation. The stable construction of the prototype is shown in a series of diagrams. Positive position feedback (PPF), integral resonant control (IRC), nonlinear integral positive position feedback (NIPPF), and negative derivative feedback (NDF) are proposed to get rid of the damaging vibration in the system. It is found that the NDF control is more efficient than other controllers for vibration suppression. The theoretical methodology is applied by using the averaging method for getting a perturbed solution. The stability and influence of various parameters of the structure are established at main and 1:1 internal resonance, which is presented as one of the worst resonance cases. Association concerning mathematical solution and computational simulation is achieved.

## Introduction

Many phenomena including photosensitive stability, electrical circuits, plasma oscillations, and buckling beams used the Duffing equation (DE)^1^. For far too long, nonlinear vibrations were of greatest significance in operative physics, engineering, applied mathematics, and in numerous real-world applications. Various methods were used to produce approximations of nonlinear oscillator solutions^2^. To produce somewhat restricted solution for the parameterized DE, the homotopy perturbation method (HPM) and Laplace transform were used^3^. The exact solution for the cubic DE, derived in this research, was demonstrated that the cubic stiffness factor and the damped variable have a destabilizing effect on the system. The controls of significantly forced systems have received a lot of attention in recent decades in a variety of practical engineering fields. In contrast to automatic frequency absorbers, which were substituted by controlling structure comprising sensors, actuators, and filters, passive oscillation absorbers have a physiological preparation related to their main organization. One important nonlinear oscillator that has been extensively noteworthy was the Van der Pol oscillator (VDP). The consequences were still being handled today. Two additional periodic forcing terms and the cubic-quintic Duffing-Van der Pol equation (DVdP) were examined^4^. The autonomous scheme accomplished linearized stability when it was close to the equilibrium locations. Moreover, stability was examined in the non-autonomous system situation by using several time scales. How to control bifurcation in a delayed extended DVdP was examined^5^. The controlled bifurcation oscillator at the perception of feedback advantage was numerically illustrated. To investigate the DVdP oscillator, the HPM was used^6^. It has been shown to be efficient and practical to compare the analytical solutions and the numerical findings. Consequently, the approach was a useful methodology for dealing with this class of nonlinear issues.

Several dynamic processes in engineering, biology, biophysics, and communications could be described by oscillators. Analytical, computational, and experimental techniques were used to study nonlinear oscillations as well as their uses in physics, chemistry, and manufacturing^7,8^. The greatest motivating nonlinear vibrations were self-excited, and it might be challenging to understand their dynamics. Researchers have extensively investigated HRVD. The structure of these oscillators has recently become a hot topic of a wealth of research. The serious analysis was done on several matters, bifurcations, limit cycle stability, hysteresis and jump phenomena, analytical solutions, plasma vibrations, and noise influence^9^. The nonlinear dynamics of ship movement were considered one of the many application areas. The roll motion has been given a lot of consideration through the years since it was the most important motion that might activate overturns. It appeared that it would continue to be popular for many years to come because so many casualties have been documented as a result of severe rolling^10^. The nonlinear characteristics of damping and restoring frequently break the motion's linearity. Hence, the two parameters were primarily responsible for adding nonlinearity to the equation. Many academics investigated a variety of roll motion models that included nonlinear components in damping and restoring. Many academics employed a variety of methodologies to pinpoint various ship behaviors^11^. The phenomena of resonance and jump amplitude, as well as consistent, chaotic, and hyper chaotic performances, were the most significant. For instance, several researchers^12,13^ explored ultra-harmonics, sub harmonics, and super harmonic oscillations in ship-rolling motion using various models. The dynamic DE behaviors were investigated^14^. Existence of both pure and impure nonlinear damping parameters and $\varphi^{6}$-equation, as well as, how they affected the system behavior has considerable bearing on the current study. Nine resonance states, of which seven were analyzed, were discovered after a thorough examination of numerous resonance states using the multiple timescale approach. An external stimulated force and the HRVD with a cubic-quintic nonlinear parameter were studied^15^. An adaptation of the Poincar'e-Lindstedt method was used to provide a roughly limited response. The numerical calculations via the Mathematica Software (MS) and the approximation solution were compared, and the results indicated good agreement.

Nonlinear vibrations included both our everyday lives and technological tools. Nonlinear oscillators were one of the most important and frequently used working prototypes in complex structures because of their importance in analyzing various nonlinear science, electrical factory production, and manufacturers. Among the most significant and well-known differential equations was the DE, whose solution was considerably relevant over recent years in physics, engineering, and environment. To improve a computational, analytical, or semi-analytical solution for this collection of issues in accordance with the DE type, many academics made a great deal of effort^16^. Furthermore, the damping DE has a stronger connection to regular life. This was why many academics have endeavored to analyze the problem. The exclusively nonlinear problem with higher-order nonlinear restoring force was theoretically and computationally analyzed^17^. The correlation between frequency and amplitude was a nonlinear oscillator key characteristic. The frequency-amplitude relationship, in this case, might be evaluated most simply using He's frequency formula^18,19^. The residual evaluation was matched to the frequency-amplitude implementation and its variations^18^. To accurately approximate a nonlinear oscillator frequency, a few advanced techniques for residual computation were described. It was suggested to make a change that added a free parameter. He's frequency-amplitude construction was used to conclude the relationship between a nonlinear oscillator frequency and amplitude using residuals from two trial solutions^19^. An example of a high nonlinearity DE was used to show how accurate the solution approach was. The most accurate and simple formulation for nonlinear oscillators was the He's frequency construction^20^. The un-damped DE and its family were effectively solved using He's frequency formulation^21^. Because of the challenges of studying the cubic DE with higher-order nonlinearity or the quadratic damping equation, this topic remains one of the most essential topics that requested extensive investigation to find more precise solutions. Because there was frequently a perfect solution to a linear equation, He's frequency construction for damped nonlinear vibration has also been a hot area of discussion. The linearized equation solution, also known as a nearly precise solution, demonstrated how to resolve the nonlinear problem. A precise solution was consistently found while linearizing equations with constant coefficients using the HPM. The current research sets out to use He's frequency construction to verify the frequency of a nonlinear vibration with linear or nonlinear dampening forces. He’s frequency was the modified periodic solution that acts as a foundation for the current inquiry. By making use of the modified HPM, the stability analysis of the perturbed pendulum motion was investigated^22^. In accordance with He’s frequency formulation, throughout the areas of fluid mechanics and dynamical system; two recent works were provided^23,24^. Vibration alleviation and energy collecting in a dynamical system of a spring-pendulum were performed^25^. The structure of the pendulum was modified using an independent electromagnetic ingathering system. The HPM straightforward and effective for many nonlinear problems; it deforms a complex problem into a linear system; however, it was still developing quickly^26^. The simplest frequency formulation for nonlinear oscillators was introduced and proved, and a modification was suggested^27^.

An attempt was made both theoretically and experimentally to preventing the oscillation on a flexible arm featuring a piezoelectric actuator^28,29^. It was seen that the NDF control was much more effective than the PPF control. A resonant control mechanism called an NDF control on a quarter-vehicle car under parametric excitation force to remove destructive vibrations was proposed^30^. The vibration behaviors of HRVD with/without of an NDF controller at main and 1:1 internal resonance situation were addressed. Additionally, the stability of the control structure was analyzed after the estimated solution that was achieved by providing the technique of multiple time scales. An NDF control for collocated structures was designed that have embedded sensors and actuators to decrease vibration levels in the system^31,32^. Furthermore, it was powerfully suggested to select the NDF controller for any oscillation attenuation after making an experimental comparison with the PPF control. An NDF controller as a novel resonant control logic which determines the feedback force using a second-order dynamic scheme, just like PPF, NPF, and the active modal tuned mass damper (AMTMD) were presented^33^. In any event, the compensator construction makes the NDF more resilient to spillover effects than other controllers. It eliminated the contribution of both the upper and lower uncontrolled modes by acting as a pass-band filter. The vibrations of the HRVD with a cubic–quintic nonlinear term and an external force via the NIPPF control was reported^15^. Moreover, at various levels of the control and structure factors, optimal operating conditions of the operation system and frequency response curves (FRCs) are described.

Given the above-mentioned aspects and the significance of nonlinear oscillation, the present paper has been motivated. Finding the various physical system behaviors while considering the HRVD is our aim. The novel strategy, often known as "new methodology" or NPA, merely converts the nonlinear ODE into a linear one. It generates a new matching frequency that looks like the linear ODE. For the advantage of the readers, a comprehensive explanation of the NPA is provided. The following is how the article is set up: The procedure for locating the analogous linear differential equation, via NPA, is described in “Description of NPA” section. The prototype HRVD and the linearization equation are numerically compared in “Methodology of Analyzing HRVD” section. This Section provides stability analysis in the absence of the excited force. The analysis of HRVD structure with NDF controller is depicted in “HRVD system with NDF controller” section. The stability analysis of HRVD with NDF controller is introduced in “Stability analysis of HRVD with NDF controller” section. The discussions and results are presented in “Discussions and results” section. The conclusion is introduced in “Conclusions” section.

## Description of NPA

In this section, the aim is to make a transformation of the given nonlinear structure to an alternative scheme that gives a differential linear equation^34^. In other words, the non-linear second order differential equation can be replaced by a linear one. The fundamental principle of the current methodology is to obtain a linearized procedure to the nonlinear form, which gives a linear oscillator covering the time collection of the vibration history^35^. It was shown that the method can overlook the complications associated with nonlinear differential equations, and the outcomes were likened with accurate solutions. The reality and exclusivity of a comprehensive corresponding linear scheme were previously inspected^36^. Now, the NPA can be described as follows:

A homogeneous third degree, in a given nonlinear differential equation, of nonlinear forces may be viewed as three quantities; the odd nonlinear damping forces, the quadratic nonlinear forces, and the restoring nonlinear odd force. Consequently, any nonlinear differential equation may be rephrased along these portions as:

1 $$ \ddot{u} + f(u\,\dot{u},\ddot{u}) + g(u\,\dot{u},\ddot{u}) + h(u\,\dot{u},\ddot{u}) = 0 $$

where $f(u\,\dot{u},\ddot{u})$ represents the odd secular terms, it signifies the Van der Pol–Rayleigh mechanism, $g(u\,\dot{u},\ddot{u})$ refers the even non-secular terms, it denotes the second-order nonlinearity of Helmholtz employment, and $h(u\,\dot{u},\ddot{u})$ indicates the odd secular terms, it specifies the cubic Duffing setup. They are addressed as:

2 $$ \left. {\begin{array}{*{20}c} {f(u,\,\dot{u},\,\ddot{u}) = a_{1} \dot{u} + b_{1} u^{2} \dot{u} + c_{1} u\dot{u}^{2} + d_{1} \dot{u}^{3} + e_{1} \ddot{u}\dot{u}^{2} } \\ {g(u,\,\dot{u},\,\ddot{u}) = a_{2} \dot{u}u + b_{2} \dot{u}^{2} + c_{2} u^{2} + d_{2} \dot{u}\ddot{u}} \\ {h(u,\,\dot{u},\,\ddot{u}) = \omega^{2} u + b_{3} u\ddot{u}\dot{u} + c_{3} \ddot{u}\dot{u}^{2} + d_{3} u^{3} + e_{3} \ddot{u}u^{2} } \\ \end{array} } \right\}, $$

where $a_{i} ,\,b_{i} ,\,c_{i} ,\,d_{i} ,\,e_{i} ,\,\,i = 1,\,2,\,3$ are some constant coefficients, and $\omega$ represents the natural frequency of the original structure.

The straightforwardness of He’s frequency^37^ may be extended to achieve theoretical formulae for the whole equivalent frequency $\Omega$ of the damping Helmholtz Rayleigh Duffing oscillator.

Newly, this issue was considered by He^38^ as consuming the characteristic of the special functions. It was recommended the following trial answer:

3 $$ u = A\cos \tilde{\Omega }t. $$

Keeping in mind the preliminary circumstances:

4 $$ u(0) = B,\,\,{\text{and}}\,\,\,\dot{u}(0) = 0. $$

To catch a modest and precise frequency–amplitude formula, a roughly corresponding linear equation of Eq. (1) will be transformed to a linear equation as:

5 $$ \ddot{u} + \tilde{\chi }\dot{u} + \Delta^{2} u = \tilde{\Lambda }. $$

Equation (5) is a corresponding linear equation and can be analyzed by the regular methods. The purpose is to use the guessing solution as given in Eq. (3). Presuming the nonappearance of a damping constant $\chi$ and the non-homogeneous part $\Lambda$, the entire frequency is condensed to equivalent frequency $\Delta$.

Following El-Dib^39–41^, the three parameters in Eq. (3) may be formulated as follows:

**Frequency integration formula**

The use of He’s formula is convenient to calculate the frequency for the advanced generalized $h(u,\,\dot{u},\,\ddot{u})$. The frequency may be calculated roughly by following El-Dib^39–41^ as:

6 $$ \Delta^{2} = \int\limits_{0}^{{2\pi /\tilde{\Omega }}} {u\,h(u,\,\dot{u},\dddot u)} dt/\int\limits_{0}^{{2\pi /\tilde{\Omega }}} {u^{2} \,dt} . $$

**Integrative damping formula**

One may calculate the frequency for specialized networks $f(u,\,\dot{u},\dddot u)$ by using He's frequency. El-Dib^39–41^ proposed the equivalence damping term as follows:

7 $$ \tilde{\chi } = \int\limits_{0}^{{2\pi /\tilde{\Omega }}} {\dot{u}\,f(u,\,\dot{u},\dddot u)} dt/\int\limits_{0}^{{2\pi /\tilde{\Omega }}} {\dot{u}^{2} \,dt} . $$

**Non-secular part**

It should be noted that the non-secular portion has the second-order formula. Therefore, Following El-Dib^39–41^, the non-homogeneity will be computed by replacing: $u \to \frac{B}{2},\,\dot{u} \to \frac{{B\tilde{\Omega }}}{2},\,\,{\text{and}}\,\,\ddot{u} \to \frac{{B\tilde{\Omega }^{2} }}{2}$.

To this end, the nonlinear Eq. (1) is transformed into the linear one as given in Eq. (4). One can utilize the normal form to Eq. (4) to estimate the stability criteria in a simpler form.

## Methodology of Analyzing HRVD

The HRVD is examined in this study in accordance with the relevance of the aforementioned processes. The standard formula of the HRVD may be written in an ordinary differential equation (ODE) as:

8 $$ \ddot{y} + \omega^{2} y + 2\mu \dot{y} + \beta_{1} y\dot{y} + \beta_{2} \dot{y}^{2} + \gamma_{1} y^{2} \dot{y} + \gamma_{2} \dot{y}^{3} + \lambda y^{3} + \delta y^{5} = F\cos \sigma t, $$

where the unknowns may be defined as follows:

**Table:** 

Symbol	Description
$y,\,\dot{y},\,\ddot{y}$	Displacement, Velocity, and Acceleration
$\omega$	Natural frequency
$\mu$	Linear damping factor
$\beta_{1}$	Impure quadratic damping factor
$\beta_{2}$	Pure quadratic damping factor
$\gamma_{1}$	Impure cubic damping factor
$\gamma_{2}$	Pure cubic damping coefficient
$\lambda$	Cubic nonlinear Duffing factor
$\delta$	Quintic nonlinear Duffing factor
$F$	External excited force
$\sigma$	External forcing coefficient

The initial conditions of Eq. (1) may be initiated as

9 $$ y(0) = A,\,\,\,{\text{and}}\,\,\,\dot{y}(0) = 0. $$

In recent decades, the majority of addressed nonlinear dynamic systems have concentrated on the perturbation theory. The overcoming of dynamic system components exhibited nonlinear behavior, and as a result, the overall structure is fundamentally nonlinear. Consequently, it is common knowledge that linear system techniques depend on the assumption that only a limited set of procedures may be performed. If the procedure range restrictions are reached, these approaches fail, which may lead to either poor performance or unstable operation. It has been recommended that these nonlinear systems can be subjected to the perturbation method. These procedures are employed to evaluate the stability and presentation of the structure as well as to generate approximate analytical solutions for these nonlinear systems. Currently, an original methodology for examining nonlinear equations in the absence of any perturbation approaches is recommended. With this sense, vast series solutions are not required, and there is no worry about their convergence. Investigating the matching linearized methodology to the nonlinear system is the main goal of this paper. In accordance with El-Dib^39–41^, the performance of a similar structure is examined by linearizing it. However, the HPM is employed to achieve the frequency response equation from the resulting linear system. It is necessary to propose a trial solution that verifies the commencement circumstances to solve the nonlinear structure by the NPA. The following description could be used to describe the recommendation trail solution:

Assuming a guessing attempt of the fundamental Eq. (1) like

10 $$ u(t) = A\cos \Omega t\,\,\,\,\,\,\,\, \Rightarrow \,\,\,\,\,\,\dot{u}(t) = - A\Omega \sin \Omega t. $$

Similar initial conditions are used, where $u(0) = A,\,\,{\text{and}}\,\,\,\dot{u}(0) = 0$, The parameter $\Omega$ refers to the total frequency, which will be determined latter.

Equation (1) may be written in an alternative form as

11 $$ \ddot{y} + f_{1} (y) + f_{2} (y,\dot{y}) + f_{3} (y,\dot{y}) = F\cos \sigma t, $$

where

12 $$ \left. {\begin{array}{*{20}c} {f_{1} (y) = \omega^{2} y + \lambda y^{3} + \delta y^{5} } \\ {f_{2} (y,\dot{y}) = 2\mu \dot{y} + \gamma_{1} y^{2} \dot{y} + \gamma_{2} \dot{y}^{3} } \\ {{\text{and}}\,\,\,\,\,f_{3} (y,\dot{y}) = \beta_{1} y\dot{y} + \beta_{2} \dot{y}^{2} } \\ \end{array} } \right\}. $$

It should be noted that the functions $f_{1} (y)$ as well as $f_{2} (y,\dot{y})$ are odd formulae; temporarily, the formulation $f_{3} (y,\dot{y})$ is an even one. He's formula can be working to regulate the frequency of the presence of odd factors in the regulatory fundamental equation of motion. El-Dib^39–41^ evaluated the frequency in such a way as before to produce:

13 $$ \mu_{eqv} = \int\limits_{0}^{2\pi /\Omega } {\dot{u}f_{2} (u,\dot{u})\,dt/\int\limits_{0}^{2\pi /\Omega } {\dot{u}^{2} dt} } = 2\mu + \frac{1}{4}\left( {\gamma_{1} + 3\Omega^{2} \gamma_{2} } \right)A^{2} , $$

14 $$ \varpi^{2} = \int\limits_{0}^{2\pi /\Omega } {uf_{1} (u)\,dt/\int\limits_{0}^{2\pi /\Omega } {u^{2} \,dt = \omega^{2} + \frac{3}{4}\lambda A^{2} + \frac{5}{8}\delta A^{4} } } , $$

and

15 $$ \Gamma = \left. {f_{3} (u,\,\dot{u})} \right|_{{u \to \frac{A}{2},\,\dot{u} \to \frac{A\Omega }{2}}} = \frac{1}{4}\left( {\beta_{1} + \Omega \beta_{2} } \right)\Omega A^{2} $$

One may now generate the matching linear equation by formulating it as

16 $$ \ddot{u} + \chi \dot{u} + \varpi^{2} u = = F\cos \sigma t - \Gamma . $$

In the absence of the frequency of the excited force ($\sigma \to 0$), Eq. (16) can be changed to its standard procedure through conversion:

17 $$ u(t) = f(t)Exp( - \chi t/2). $$

Therefore, it can be expressed as:

18 $$ \ddot{f}(t) + \Omega^{2} f(t) = (F - \Gamma )Exp(\mu_{eqv} t/2), $$

where $$\Omega = \sqrt {\varpi^{2} - \chi^{2} /4}$$.

Assuming similar previous preliminary conditions,$f(0) = A,\,\,\,{\text{and}}\,\,\,\dot{f}(0) = 0$, and returning to the previous linear Eq. (16), the solution of the linear equation ($\sigma \to 0$)can be formulated as:

19 $$ u(t) = \left( {C_{1} \cos \Omega t + C_{2} \sin \Omega t} \right)Exp( - \chi t/2) + \frac{1}{{\Omega^{2} }}(F - \Gamma ), $$

where

20 $$ C_{1} = A - \frac{1}{{\Omega^{2} }}(F - \Gamma )\,\,\,{\text{and}}\,\,\,\,\,\,\,\,\,C_{2} = \frac{\mu \chi }{{2\Omega }}C_{1} . $$

The stability criteria, in case of $\sigma \to 0$, can be expressed as:

21 $$ \Omega^{2} > 0,\,\,\,\,\,{\text{and}}\,\,\,\,\,\chi > 0. $$

Returning to the basic equation given by Eq. (8), the NPA enables us to create identical initial conditions (9), thereby being equivalent to the linear equation as shown in Eq. (16). The consequences of the corresponding dampening term as given in Eq. (13) and comparable frequency as given in Eq. (14) were discussed before. It is interesting to examine the relationship involving the linear ODE solution (non-perturbative solution) and the computation solution of the previous Eq. (14) using the numerical calculations via the MS. Therefore, the subsequent numbers for the applied settings are considered.

$$ \omega = 1.0,\,\mu = 0.7,\,\beta_{1} = 1.2,\,\beta_{2} = 1.5,\,\gamma_{1} = 1.3,\,\gamma_{2} = 1.4,\,\lambda = 1.6,\,\delta = 2.5,\,F = 0.2,\,\sigma = 0.1,\,\,\,{\text{and}}\,\,A = 0.1 $$

**Figure 1:**
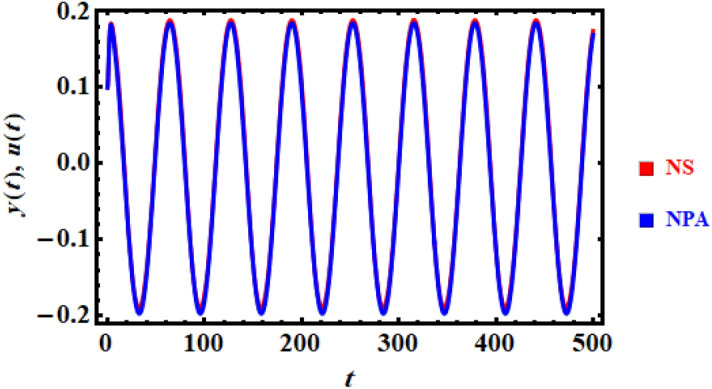
Shows an association concerning the solutions of HRVD and its alternative linear equation.

In light of the above sample chosen system, the command FindRoot through the MS produces the value of the total frequency as: $\Omega = 0.71838$. A comparison between the associated linear ODE equation and the computation solution of the fundamental Eq. (8) created the numerical calculation is also useful. The non-perturbative equation is given by Eq. (16). The framework is shown in this comparison, as shown in Fig. 1. The figure is obtained considering the prior data for an adequate sample with the given details. It involves the two equations as well. As can be seen, the findings are generally consistent with one another. Additionally, the MS shows that, up to a time of 500 units, the absolute difference concerning the theoretical and computational findings is 0.0132417.

As previously shown, the new methodology reveals an equivalent equation, which enables us to examine the stability configuration of the original prototype. Therefore, the criteria that arise in Eq. (16) can be represented together with the original equation as shown in Eq. (8).

$$ \mu = 0.5,\,\gamma_{1} = 1.3,\,\gamma_{2} = 2.0,\,\lambda = 016,\,\delta = 2.5,\,\lambda = 1.6,\,\,\,{\text{and}}\,\,\delta = - 2.5, $$

**Figure 2:**
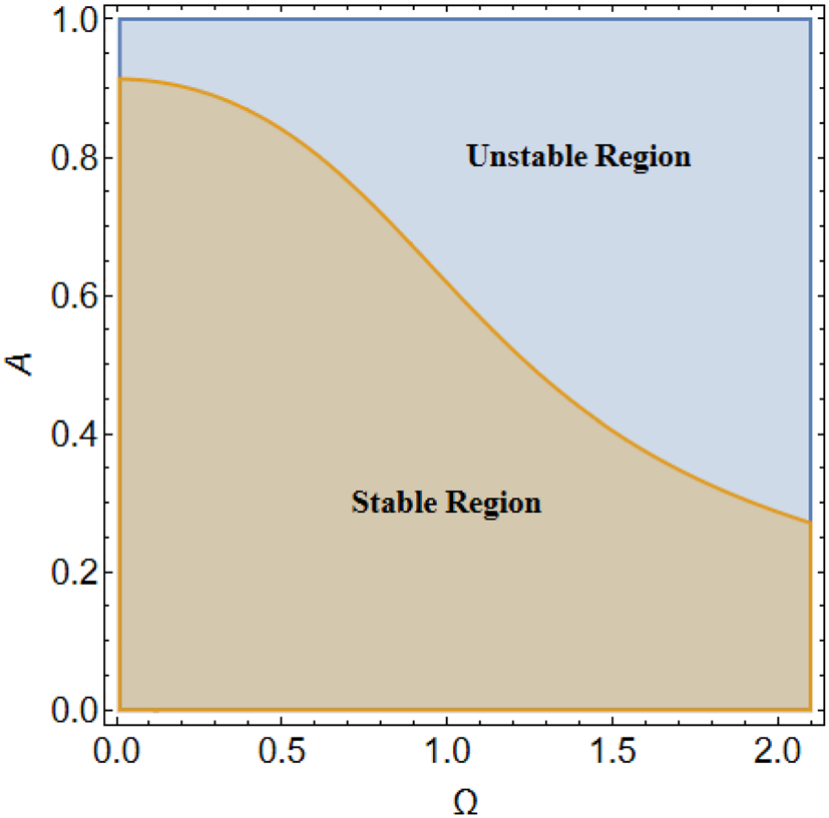
Depicts the stable/unstable regions.

It is convenient to use the MS to diagram the stability picture by graphing the initial amplitude $A$ versus the total frequency $\Omega$ as revealed in Fig. 2.

**Figure 3:**
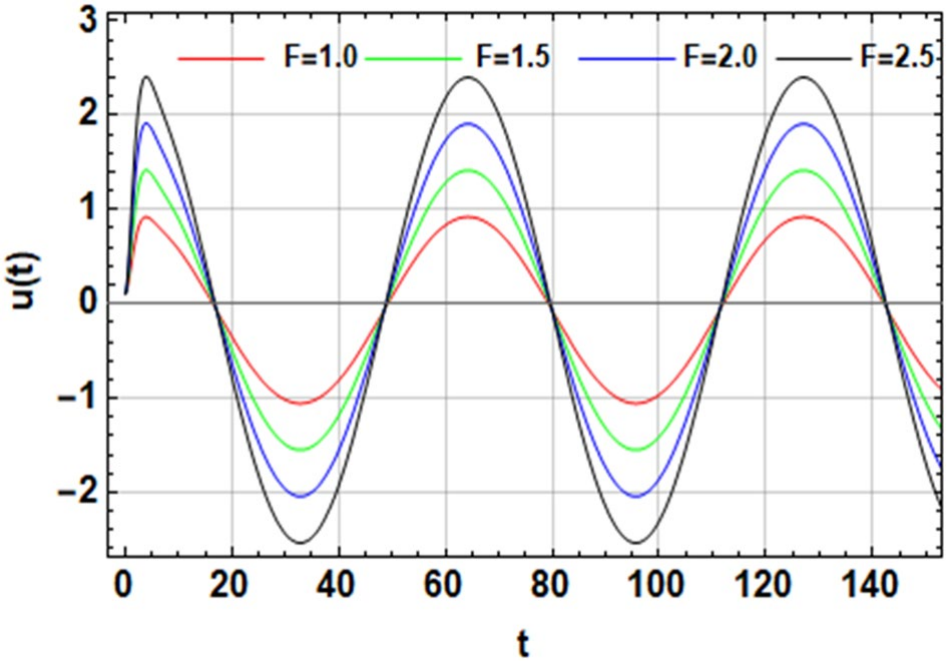
Portrays the impact of $F$.

**Figure 4:**
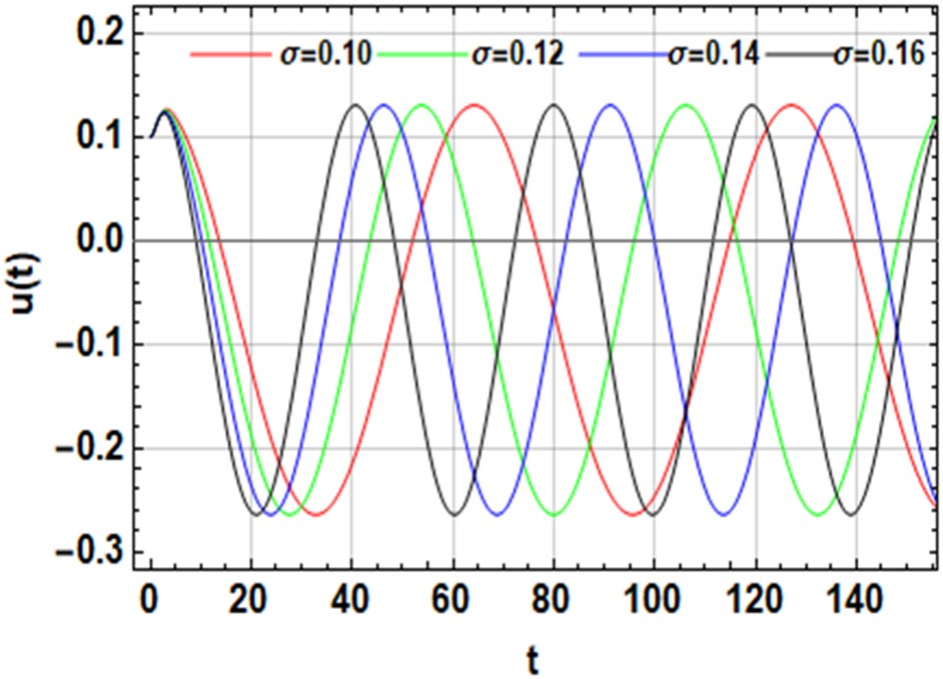
Shows the inspiration of $\sigma$.

**Figure 5:**
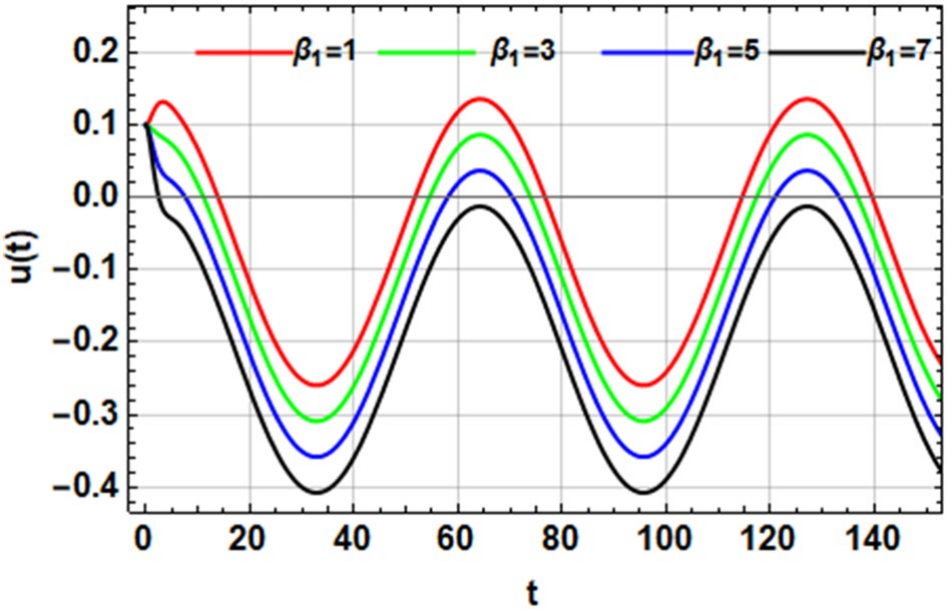
Depicts the stimulus of $\beta_{1}$.

**Figure 6:**
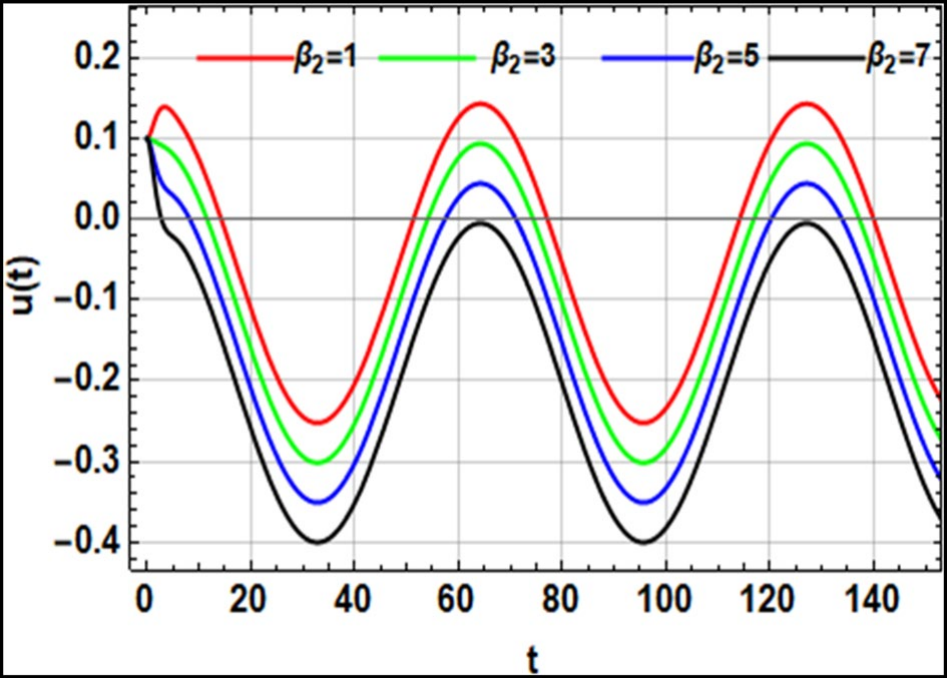
Represents the effect of $\beta_{2}$.

On the other hand, the right-hand side of the equivalent Eq. (16) has no implication on the stability diagram as shown in Fig. 2. Therefore, the influences of the parameters $F,\,\sigma ,\,\beta_{1} ,\,\,{\text{and}}\,\,\beta_{2}$ are depicted throughout Figs. 3, 4, 5 and 6 to depict the role of each of them in the amplitudes of the solution of the wave solutions of Eq. (19). Subsequently, in what follows, Fig. 3 shows the influence of the excited force $F$. As shown, the increase in $F$ increases the solution amplitude. Therefore, one can say that the parameter $F$ has a destabilizing impact on the stability profile. Once more, we show the NPA provided by Eq. (16). For different values of the external forcing coefficient $\sigma$, Fig. 4 plots the distribution of the time-dependent function versus time $t$. The distribution function is depicted as a periodic solution. Consequently, as $\sigma$ grows, the wave solution amplitude remains fixed. In addition, as $\sigma$ is raised, the wavelength decreases. In other words, the horizontal $t -$ axis has two zeros moving in the direction of enhancing values. These findings demonstrate the stabilizing effect on the stability configuration. Figure 5 depicts the impact of the impure quadratic damping coefficient $\beta_{1}$. As recognized, the solution amplitude decreases according to the increase in $\beta_{1}$. Hence, it can be claimed that $\beta_{1}$ has a destabilizing effect on the stability picture. The effect of the pure quadratic damping coefficient $\beta_{2}$ is shown in Fig. 6. It is common knowledge that depending on the rising of $\beta_{2}$ decreases the solution amplitude. Consequently, it can be said that $\beta_{2}$ destabilizes the stability profile.

**Figure 7:**
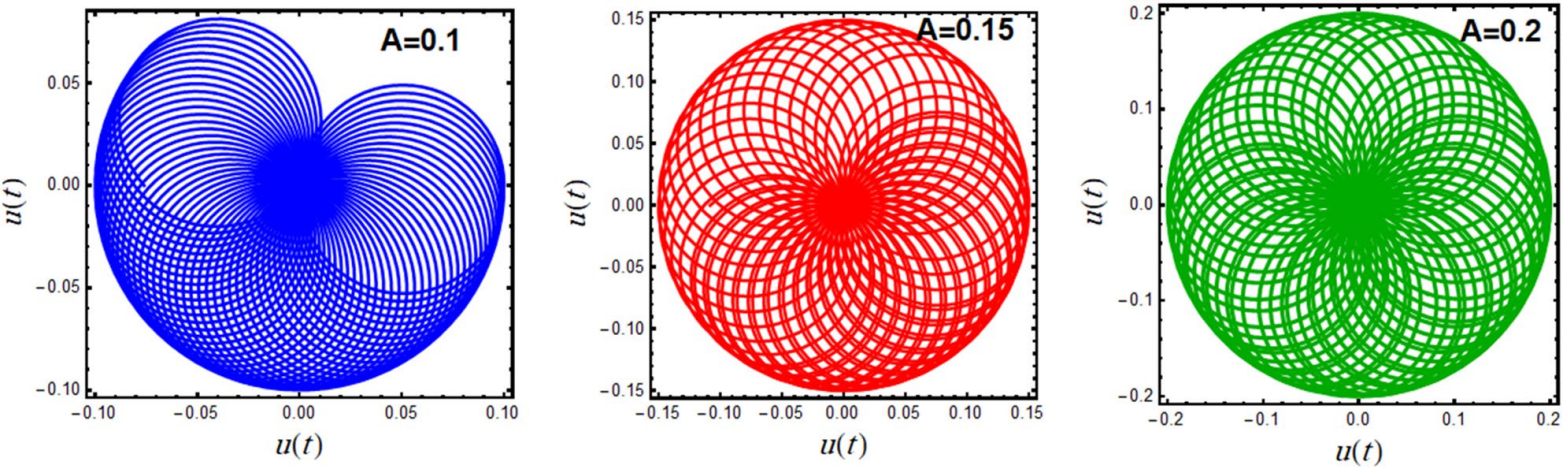
Displays the polar plots of the linear equivalent differential (19) for the variation of the initial amplitude $A$.

**Figure 8:**
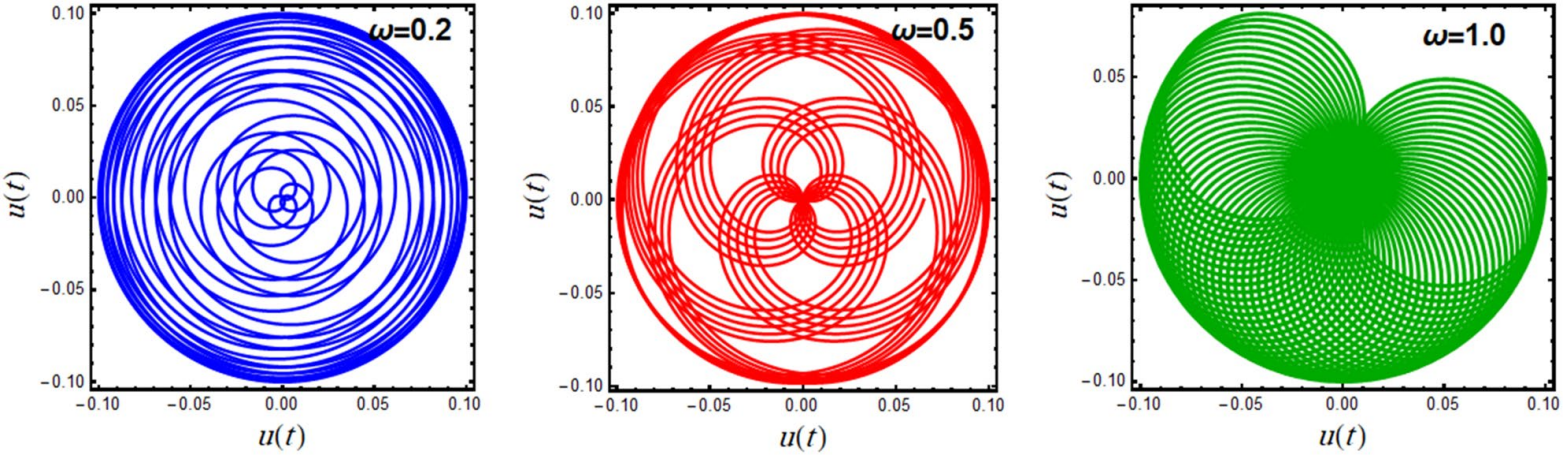
Displays the polar plots of the linear equivalent differential (19) for the variation of the natural frequency $\omega$.

A polar plot of Eq. (19) is designed in light of the parameters**’** values for $A = 0.1,\,0.15,\,0.2$ and $\omega = 0.1,\,0.5,\,1.0$, as shown in Figs. 7 and 8, correspondingly. These diagrams obtain the performance of the corresponding solution when a small change in its initial situation and its essential natural frequency is considered. It is found that the designed curves in these figures rotate in repeated paths, forming these wonderful spiral shapes, which are symmetrical around its center. It is found that this symmetry increases with increasing $A$, as seen in Fig. 7, and the reverse is true with increasing $\omega$, as shown in Fig. 8. These centers are seen as the accumulative point of these curves which varies along with the nominated values of $A$ and $\omega$. These curves rotate in several arrangements of closed or semi closed elliptical paths originating from this accumulative central point. The numeral of these curves declines and grows, with the increase of the amounts of $A$ and $\omega$, respectively. As seen from these figures, one can observe that the first graph of Fig. 7 is similar to the last one of Fig. 8 due to the utilizing the same data in these two plots.

**Figure 9:**
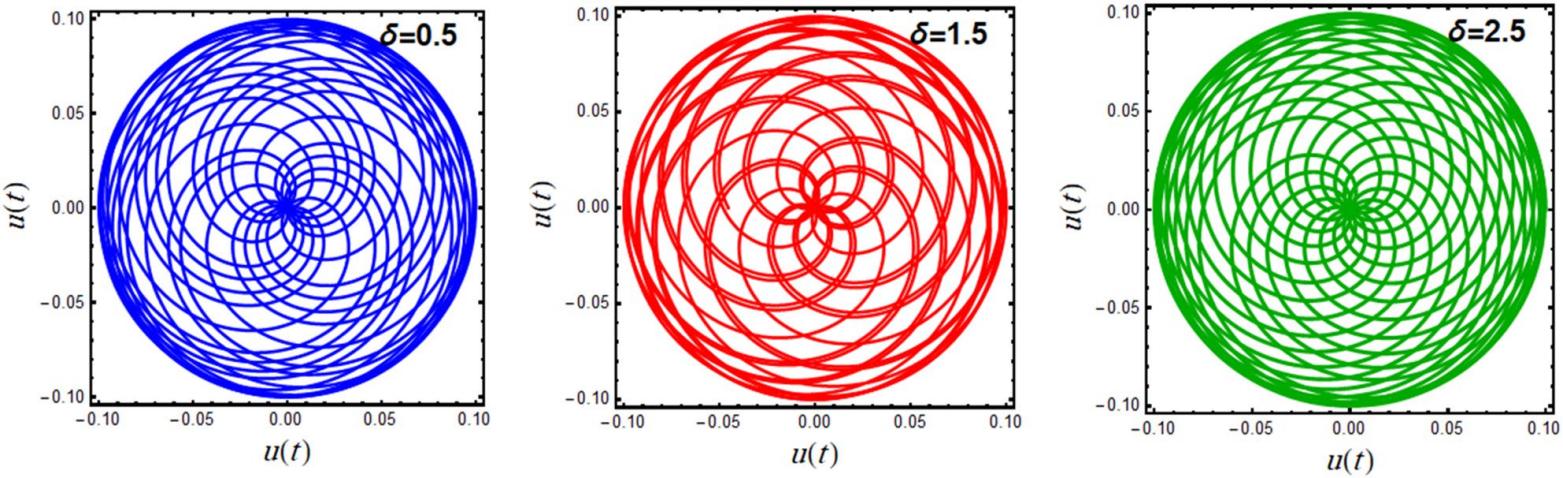
Displays the polar plots of the linear equivalent differential (19) for the variation of the parameter $\delta$.

**Figure 10:**
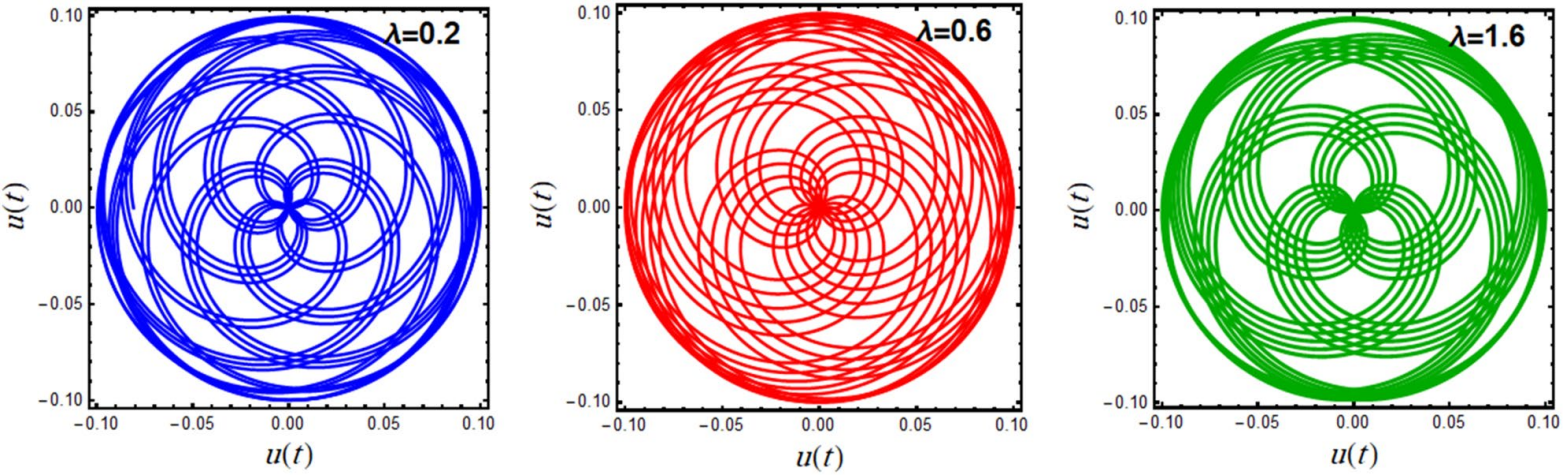
Displays the polar plots of the linear equivalent differential (19) for the variation of the parameter $\lambda$.

Through the time interval $\left[ {0,\,50\pi } \right]$, Figs. 9 and 10 have been plotted to illustrate the function $u(t)$ in a polar form according to the various values of $\delta = 0.5,\,1.5,\,2.5$*,* and $\lambda = 0.2,\,0.6,\,1.6$, respectively. This simulation is represented by circulated intersected curves and distributed symmetrically about their centers. That distribution provides an initiation around the stable mode in which these curves act. The circulation of these interconnected curves rises or declines based on the impact of the affected factors. It is found that when the amounts of $\delta$ rise, the circulations rises, as exposed in the graphs of Fig. 9. The opposite holds true for portions of Fig. 10 with the growth of $\lambda$ values. Furthermore, it can be seen that the concentration and wave wideness of these curves rise with the rise of $\lambda$ and decreases with the rise of $\delta$, which implies that the influence of $\lambda$ is directly proportional, while the influence of $\delta$ is inversely proportional to the curves’ density and thickness. It is obvious that the two second plots of Figs. 9 and 10 are similar due to the similarity of the usage data.

## HRVD system with NDF controller

In the current section, we present a comprehensive explanation of the method of reducing the resulting vibrations of Eq. (8) as shown in Fig. 1, which describes the HRVD system with cubic-quintic nonlinear terms subjected to the external excitation force. Accordingly, we apply the NDF control technique as previously demonstrated^28–33^ to the fundamental equation of motion (8). Therefore, the considered system can be formulated after control to become:

Applying the NDF control as previously demonstrated^28–33^ to the fundamental equation of motion (8), it may be formulated in the following system:

22 $$ \ddot{y} + \omega^{2} y + 2\varepsilon \mu \dot{y} + \varepsilon \beta_{1} y\dot{y} + \varepsilon \beta_{2} \dot{y}^{2} + \varepsilon \gamma_{1} y^{2} \dot{y} + \varepsilon \gamma_{2} \dot{y}^{3} + \varepsilon \lambda y^{3} + \varepsilon \delta y^{5} = \varepsilon F\cos \sigma t + \varepsilon \,G_{1} \dot{u}, $$

23 $$ \ddot{u} + \omega_{1}^{2} u + 2\varepsilon \mu_{1} \,\dot{u} = - \varepsilon \,G_{2} \dot{y}, $$

where the parameters of NDF controller system are presented as follows:

$u$ is displacement, $\omega_{1}$ is the normal frequency, $\mu_{1}$ is the linear damping coefficient, $G_{1}$ and $G_{2}$ are the signals gain from NDF controller.

The calculation of RK-4 is used to illustrate the time history graph figure and the phase portrait before and after merging the NDF controller at one of the worst resonance cases which presented as the simultaneous primary and 1:1 internal resonance ($\sigma \approx \omega$ and $\omega_{1} \approx \omega$). These achieved computations are based on MATLAB® Software controller. Accordingly, the model selected scheme is:

$$ \omega = 1.0,\,\mu = 0.7,\,\beta_{1} = 1.2,\,\beta_{2} = 1.5,\,\gamma_{1} = 1.3,\,\gamma_{2} = 1.4,\,\lambda = 1.6,\,\delta = 2.5,\,F = 0.2,\mu_{1} = 0.0007,\,G_{1} = 0.8,G_{2} = 0.8\, $$

**Figure 11:**
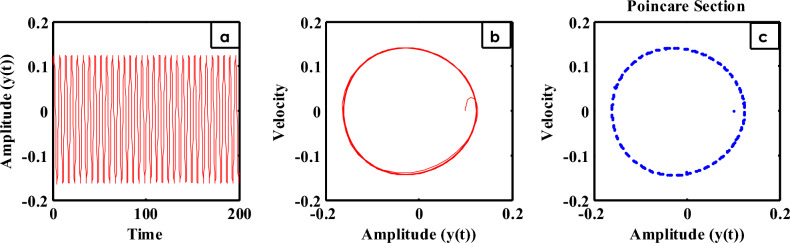
Time history, phase portrait and Poincare section of style deprived of control at $\sigma = \omega$.

**Figure 12:**
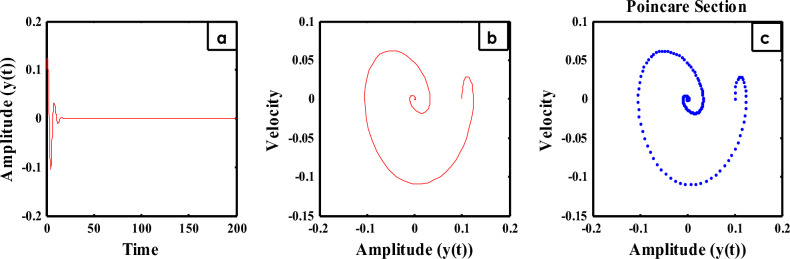
Time history, phase portrait and Poincare section of controlled style at $\sigma = \omega$ and $\omega_{1} = \omega$.

The time history is shown in Fig. 11a for the steady state amplitude of HRVD model before applying the controller. As revealed, the amplitude scopes 0.1235. Instantaneously, Fig. 11b characterizes the phase portrait concerning the velocity and amplitude for the similar situation, which displays the chaotic attractor and estimated multi-limit cycle. In accumulation, the reaction of the HRVD model through the NDF control is portrayed as a Poincare map drawn in Fig. 11c that specifies the motion’s type of the model and the control. On the opposite side, Fig. 12a represents the amplitude of the deliberated construction after merging the NDF controller. It is found that the amplitude develops 0.0006373. Consequently, conferring to this control, the amplitudes have been condensed by the ratio 99.48%. Furthermore, Fig. 12b and c show the phase portrait between the velocity and amplitude and a Poincare map diagram after applying the NDF control, which displays enhancement of the chaotic attractor and the limited cycle numerals. Lastly, the efficiency of the NDF control $E_{a}$ is addressed as ($E_{a} =$ steady-state amplitude of the construction beforehand NDF divided by afterward controlling) and is of 193.79. Analogous outcomes have been achieved in our preceding study^28–33^.

**Figure 13:**
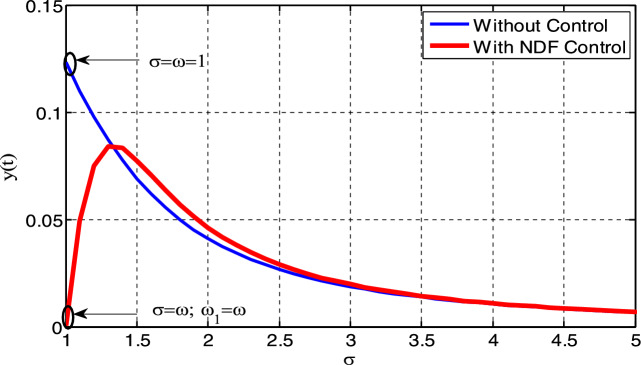
Diagram response to attain the measured one of worst resonance cases of the structure before and after the NDF controller.

Furthermore, Fig. 13 demonstrates that the highest steady-state amplitude occurs in one of the worst resonance cases (the primary resonance) before the inclusion of the controller. However, after the inclusion of the NDF controller, the system amplitude appears to be diminished when the controller operates when the primary resonance and 1:1 internal resonance are together, which leads to the quality of the NDF controller on the system.

Applying the averaging technique as mentioned in^42–45^ to get the frequency response equations, the overall solution of Eqs. (22) and (23) when $\varepsilon = 0$ is expressed as follows:

24 $$ y = a_{1} \,\cos (\omega t + \phi_{1} ), $$

25 $$ u = a_{2} \,\cos (\omega_{1} t + \phi_{2} ), $$

where $a_{1}$, $a_{2}$,$\phi_{1}$ and $\phi_{2}$ are constants. The derivative of the previous equations with respect to $t$ yields

26 $$ \dot{y} = - \omega \,a_{1} \,\sin (\omega t + \phi_{1} ), $$

27 $$ \dot{u} = - \omega_{1} \,a_{2} \,\sin (\omega_{1} t + \phi_{2} ). $$

On the other hand, when $\varepsilon \ne 0$ but nevertheless small enough, $a_{1}$, $a_{2}$, $\phi_{1}$ and $\phi_{2}$ are assumed as formulations of time in Eqs. (24) and (25). Thus, the derivative of these equations with respect to $t$ yields

28 $$ \dot{y} = - \left( {\omega + \dot{\phi }_{1} } \right)\,a_{1} \,\sin (\omega t + \phi_{1} )\, + \dot{a}_{1} \,\cos (\omega t + \phi_{1} ), $$

29 $$ \dot{u} = - \left( {\omega_{1} + \dot{\phi }_{2} } \right)\,a_{2} \,\sin (\omega_{1} t + \phi_{2} )\, + \dot{a}_{2} \,\cos (\omega_{1} t + \phi_{2} ). $$

Comparing Eqs. (26) and (27) with Eqs. (28) and (29) gives the following:

30 $$ - \dot{\phi }_{1} \,a_{1} \,\sin (\omega t + \phi_{1} )\, + \dot{a}_{1} \,\cos (\omega t + \phi_{1} ) = 0, $$

31 $$ - \dot{\varphi }_{2} \,a_{2} \,\sin (\omega_{1} t + \varphi_{2} )\, + \dot{a}_{2} \,\cos (\omega_{1} t + \varphi_{2} ) = 0. $$

Differentiating Eqs. (28) and (29) with regard to $t$ yields

32 $$ \ddot{y} = - \omega \,\dot{a}_{1} \,\sin (\omega t + \phi_{1} )\, - \omega \left( {\omega + \dot{\phi }_{1} } \right)a_{1} \,\cos (\omega t + \phi_{1} ), $$

33 $$ \ddot{u} = - \omega_{1} \,\dot{a}_{2} \,\sin (\omega_{1} t + \phi_{2} )\, - \omega_{1} \left( {\omega_{1} + \dot{\phi }_{2} } \right)a_{2} \,\cos (\omega_{1} t + \phi_{2} ). $$

Substituting from Eqs. (24)–(27), (32) and (33) into Eqs. (22) and (23), one obtain the following equations

34 $$ \left\{ {\left. \begin{gathered} - \omega \,\dot{a}_{1} \,\sin (\omega t + \phi_{1} )\, - \omega \left( {\omega + \dot{\phi }_{1} } \right)a_{1} \,\cos (\omega t + \phi_{1} ) \hfill \\ + \omega^{2} a_{1} \,\cos (\omega t + \phi_{1} ) - 2\varepsilon \mu \omega \,a_{1} \,\sin (\omega t + \phi_{1} ) \hfill \\ - \varepsilon \beta_{1} \omega a_{1}^{2} \,\cos (\omega t + \phi_{1} )\sin (\omega t + \phi_{1} ) + \varepsilon \beta_{2} \omega^{2} \,a_{1}^{2} \,\sin^{2} (\omega t + \phi_{1} ) \hfill \\ - \varepsilon \gamma_{1} \omega a_{1}^{3} \,\cos^{2} (\omega t + \phi_{1} )\sin (\omega t + \phi_{1} ) - \varepsilon \gamma_{2} \,\omega^{3} a_{1}^{3} \,\sin^{3} (\omega t + \phi_{1} ) \hfill \\ + \varepsilon \lambda a_{1}^{3} \,\cos^{3} (\omega t + \phi_{1} ) + \varepsilon \delta \,a_{1}^{5} \,\cos^{5} (\omega t + \phi_{1} ) \hfill \\ \end{gathered} \right\} = } \right.\varepsilon F\cos \sigma t + \varepsilon G_{1} \omega_{1} a_{2} \,\sin (\omega_{1} t + \phi_{2} ), $$

35 $$ \left\{ \begin{gathered} - \omega_{1} \,\dot{a}_{2} \,\sin (\omega_{1} t + \phi_{2} )\, - \omega_{1} \left( {\omega_{1} + \dot{\phi }_{2} } \right)a_{2} \,\cos (\omega_{1} t + \phi_{2} ) \hfill \\ + \omega_{1}^{2} a_{2} \,\cos (\omega_{1} t + \phi_{2} ) - 2\varepsilon \mu_{1} \,\omega_{1} \,a_{2} \,\sin (\omega_{1} t + \phi_{2} ) \hfill \\ \end{gathered} \right\} = \varepsilon G_{2} \omega \,a_{1} \,\sin (\omega t + \phi_{1} ). $$

The concurrent main and 1:1 internal resonance ($\sigma \approx \omega$ and $\omega_{1} \approx \omega$) are studied in this work. Now, the averaging equations are gotten by using the detuning parameters ($\sigma_{1}$, $\sigma_{2}$) according to ($\sigma = \omega + \varepsilon \sigma_{1}$, $\omega_{1} = \omega + \varepsilon \sigma_{2}$). Inserting Eqs. (30) and (31) into Eqs. (34) and (35), then using the averaging equations of $\dot{a}_{m}$ and $\dot{\phi }_{m}$ as demonstrated in^31,32^, one obtains

36 $$ \dot{a}_{1} = - \varepsilon \mu a_{1} - \frac{1}{8}\varepsilon \gamma_{1} a_{1}^{3} - \frac{3}{8}\varepsilon \gamma_{2} \omega^{2} a_{1}^{3} + \frac{1}{2\omega }\varepsilon F\sin \theta_{1} + \frac{1}{2\omega }\varepsilon G_{1} \omega_{1} a_{2} \cos \theta_{2} , $$

37 $$ \dot{\phi }_{1} a_{1} = \frac{3}{8\omega }\varepsilon \lambda a_{1}^{3} + \frac{5}{16\omega }\varepsilon \delta a_{1}^{5} - \frac{1}{2\omega }\varepsilon F\cos \theta_{1} + \frac{1}{2\omega }\varepsilon G_{1} \omega_{1} a_{2} \sin \theta_{2} , $$

38 $$ \dot{a}_{2} = - \varepsilon \mu_{1} a_{2} - \frac{1}{{2\omega_{1} }}\varepsilon G_{2} \omega a_{1} \cos \theta_{2} , $$

39 $$ \dot{\phi }_{2} a_{2} = \frac{1}{{2\omega_{1} }}\varepsilon G_{2} \omega a_{1} \sin \theta_{2} . $$

where $\theta_{1} = \varepsilon \sigma_{1} t - \phi_{1}$ and $\theta_{2} = \varepsilon \sigma_{2} t + \phi_{2} - \phi_{1}$. Thus $\dot{\theta }_{1} = \varepsilon \sigma_{1} - \dot{\dot{\phi }}_{1}$ and $\dot{\theta }_{2} = \varepsilon (\sigma_{2} - \sigma_{1} ) + \dot{\theta }_{1} + \dot{\dot{\phi }}_{2}$ yields

40 $$ \dot{\theta }_{1} = \varepsilon \sigma_{1} - \frac{3}{8\omega }\varepsilon \lambda a_{1}^{2} - \frac{5}{16\omega }\varepsilon \delta a_{1}^{4} + \frac{1}{{2\omega a_{1} }}\varepsilon F\cos \theta_{1} - \frac{1}{{2\omega a_{1} }}\varepsilon G_{1} \omega_{1} a_{2} \sin \theta_{2} , $$

41 $$ \dot{\theta }_{2} = \varepsilon (\sigma_{2} - \sigma_{1} ) + \dot{\theta }_{1} + \frac{1}{{2\omega_{1} a_{2} }}\varepsilon G_{2} \omega a_{1} \sin \theta_{2} . $$

Equations (36), (38), (40) and (41) are addressed as autonomous amplitude-phase modulating equations.

## Stability analysis of HRVD with NDF controller

By considering $\dot{a}_{m} = \dot{\theta }_{m} = 0$, where ($m = 1,2$), the fixed points can be obtained as mentioned in^46,47^. Here, the steady-state solution of the system over the NDF control related to the fixed points as specified in Eqs. (36)–(39) is accomplished.

42 $$ \mu a_{1} + \frac{1}{8}\gamma_{1} a_{1}^{3} + \frac{3}{8}\gamma_{2} \omega^{2} a_{1}^{3} = \frac{1}{2\omega }F\sin \theta_{1} + \frac{1}{2\omega }G_{1} \omega_{1} a_{2} \cos \theta_{2} , $$

43 $$ \sigma_{1} a_{1} - \frac{3}{8\omega }\lambda a_{1}^{3} - \frac{5}{16\omega }\delta a_{1}^{5} = - \frac{1}{2\omega }F\cos \theta_{1} + \frac{1}{2\omega }G_{1} \omega_{1} a_{2} \sin \theta_{2} , $$

44 $$ \mu_{1} a_{2} = - \frac{1}{{2\omega_{1} }}G_{2} \omega a_{1} \cos \theta_{2} , $$

45 $$ (\sigma_{2} - \sigma_{1} )a_{2} = - \frac{1}{{2\omega_{1} }}G_{2} \omega a_{1} \sin \theta_{2} . $$

Then, the frequency response equations inside these fixed solutions are obtained as follows:

46 $$ \left( {\sigma_{1} a_{1} - \frac{{3\lambda a_{1}^{3} }}{8\omega } - \frac{{5\delta a_{1}^{5} }}{16\omega } - \frac{{(\sigma_{1} - \sigma_{2} )G_{1} \omega_{1}^{2} a_{2}^{2} }}{{G_{2} \omega^{2} a_{1} }}} \right)^{2} + \left( {\mu a_{1} + \frac{{\gamma_{1} a_{1}^{3} }}{8} + \frac{{3\gamma_{2} \omega^{2} a_{1}^{3} }}{8} + \frac{{\mu_{1} G_{1} \omega_{1}^{2} a_{2}^{2} }}{{G_{2} \omega^{2} a_{1} }}} \right)^{2} = \frac{{F^{2} }}{{4\omega^{2} }}, $$

47 $$ \left( {\mu_{1}^{2} + (\sigma_{1} - \sigma_{2} )^{2} } \right)\,a_{1}^{2} = \frac{{G_{2}^{2} \omega^{2} a_{1}^{2} }}{{4\omega_{1}^{2} }}. $$

To establish the stability pattern of the steady-state solution, assume the following prospects:

48 $$ a_{m} = a_{m\,0} + a_{m\,1} ,\theta_{m} = \theta_{m\,0} + \theta_{m\,1} ,(m = 1,\,2), $$

where $a_{m\,0}$ and $\theta_{m\,0}$ are the solutions of Eqs. (36), (38), (40) and (41); the actual minor and the disturbed amounts are addressed by $a_{m1}$ and $\theta_{m1}$. Replacing Eq. (48) with Eqs. (36), (38), (40) and (41), while maintaining only the linear terms of $a_{m\,1}$ and $\theta_{m\,1}$, one obtains the equation:

49 $$ \left[ {\begin{array}{*{20}c} {\dot{a}_{11} } \\ {\dot{\theta }_{11} } \\ {\dot{a}_{21} } \\ {\dot{\theta }_{21} } \\ \end{array} } \right] = \left[ {\begin{array}{*{20}c} {r_{11} } & {r_{12} } & {r_{13} } & {r_{14} } \\ {r_{21} } & {r_{22} } & {r_{23} } & {r_{24} } \\ {r_{31} } & {r_{32} } & {r_{33} } & {r_{34} } \\ {r_{41} } & {r_{42} } & {r_{43} } & {r_{44} } \\ \end{array} } \right]\,\left[ {\begin{array}{*{20}c} {a_{11} } \\ {\theta_{11} } \\ {a_{21} } \\ {\theta_{21} } \\ \end{array} } \right], $$

herein the overhead square matrix is called the Jacobian matrix ${\rm X}$. The coefficients amounts $r_{i\,j}$, ($i,j = 1,2,3,4$) are recorded in the Appendix. Consequently, the eigenvalues $\gamma_{r}$, ($r = 1,2,3,4$) are specified by ${\rm X} - \gamma_{r} \,I_{4 \times 4} = 0$ and give the following equation:

50 $$ \gamma^{4} + R_{1} \,\gamma^{3} + R_{2} \,\gamma^{2} + R_{3} \,\gamma + R_{4} = 0, $$

where the coefficients $R_{1}$, $R_{2}$, $R_{3}$ and $R_{4}$ are recognized from the background. In view of the Routh–Hurwitz standard^43–45^, if the real part of the eigenvalue is negative, then the periodic solution is stable; if not, it is unstable.

## Discussions and results

### Frequency response curve (FRC) and impact of various parameters

In the subsequent Figs. 14, 15, 16, 17, 18, 19, 20, 21, 22, 23, 24, 25 and 26, it must be noticed that the solid curves refer to the stable zones, whereas the dashed ones characterize the unstable areas.

**Figure 14:**
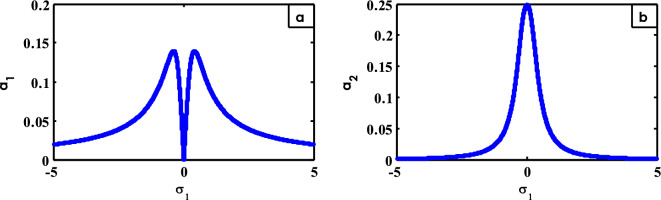
FRC of NDF controlled system at $\sigma_{2} = 0$ (**a**) ($a_{1}$ against $\sigma_{1}$) and (**b**) ($a_{2}$ against $\sigma_{1}$).

**Figure 15:**
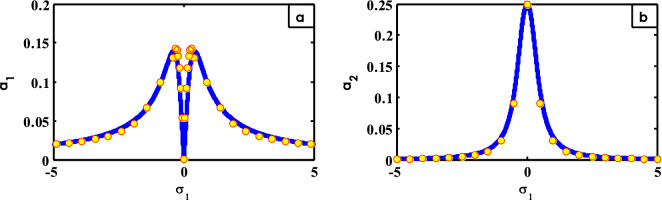
FRC contrast concerning both RK-4 (with circle) and theoretical solution (with line).

**Figure 16:**
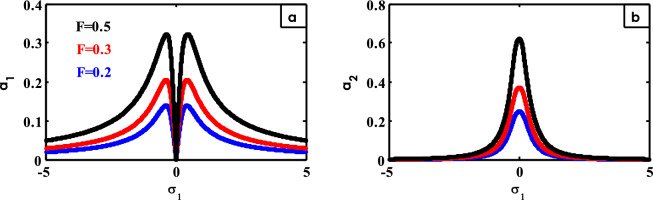
Effects of $F$ on FRC.

**Figure 17:**
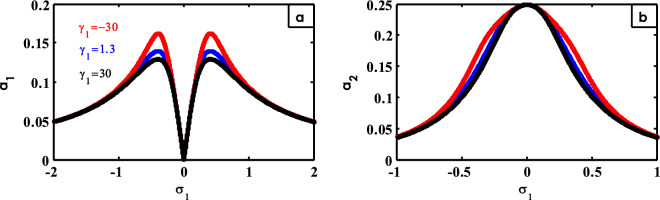
Effects of $\gamma_{1}$ on FRC.

**Figure 18:**
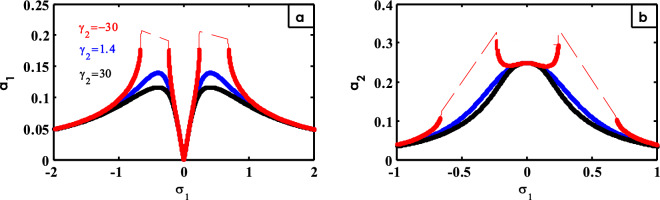
Effects of $\gamma_{2}$ on FRC.

**Figure 19:**
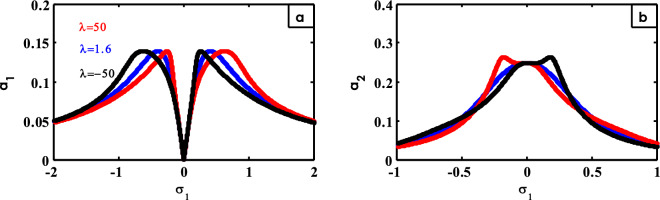
Effects of $\lambda$ on FRC.

**Figure 20:**
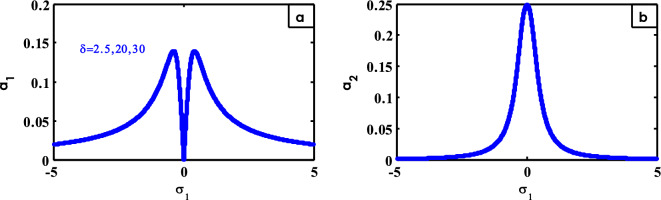
Effects of $\delta$ on FRC.

**Figure 21:**
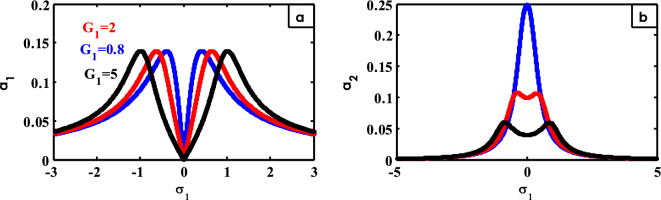
Effects of $G_{1}$ on FRC.

**Figure 22:**
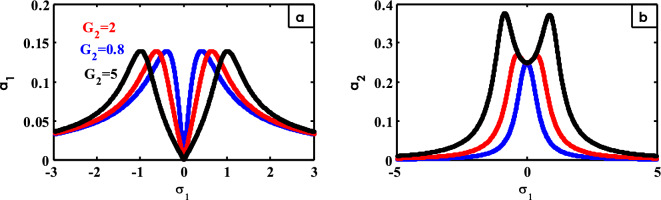
Effects of $G_{2}$ on FRC.

**Figure 23:**
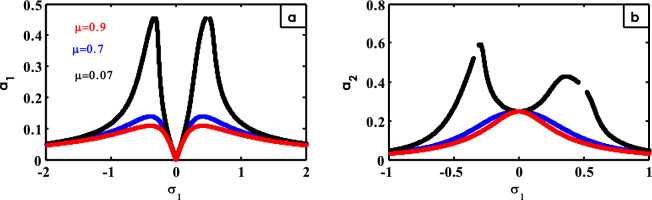
Effects of $\mu$ on FRC.

**Figure 24:**
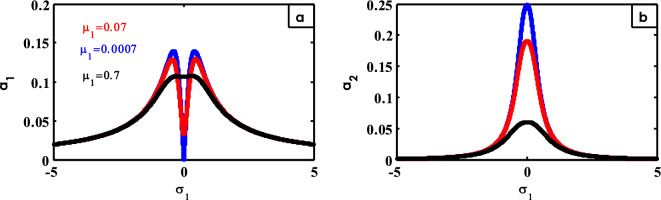
Effects of $\mu_{1}$ on FRC.

**Figure 25:**
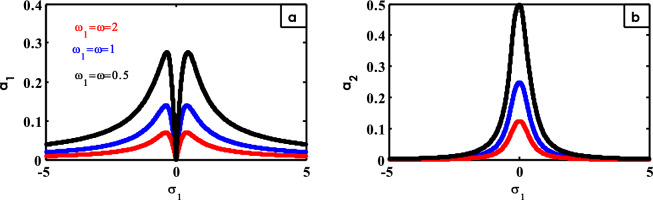
Effects of $\omega$ on FRC.

**Figure 26:**
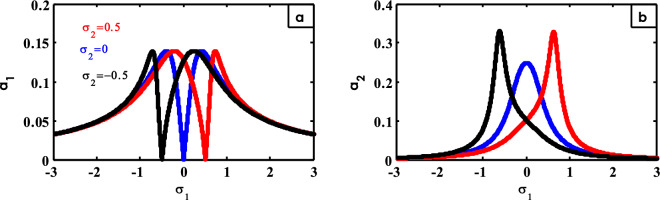
Effects of $\sigma_{2}$ on FRC.

The FRC for both the model amplitude $a_{1}$ and the amplitude of the related NDF controller are exposed in Fig. 14. For this aim, Fig. 14a shows the amplitude prototypical $a_{1}$ versus $\sigma_{1}$. Instantaneously, Fig. 14b represents the control amplitude $a_{2}$ against $\sigma_{1}$. Figure 14a has two peaks on two sides of $\sigma_{1} = 0$ and region between these peaks is called vibration suppression bandwidths.

Furthermore, it seems from Fig. 14a that the oscillations are damped in the zone of the frequency bandwidth after using the NDF control. Subsequently, we may indicate that the optimal mode of oscillation lessening is $\sigma_{1} = \sigma_{2} = 0$. Previous instances were involved^15,30,42,48^.

To explain the verification of the mathematical procedures and calculation methodologies, a comparison between them will be made. For this purpose, the contrast of FRC concerning both analytic solution in the solid lines and numerical outcomes in circles is shown in Fig. 15. It is shown that from this figure and from both Tables 1 and 2 that the analytical solution is merely reliable with the calculation path as shown earlier^15,30,42,48^.

**Table 1:** A comparison concerning RK-4 and averaging solutions ($a_{1}$ by $\sigma_{1}$).

$\sigma_{1}$	Numerical calculation	Averaging solution	Absolute error
− 4.9	0.02028	0.020334249400201	5.42494E−05
− 4.4	0.0217	0.022625531319440	0.000925531
− 3.9	0.02359	0.025493211859700	0.001903212
− 3.4	0.02624	0.029188950279256	0.00294895
− 2.9	0.03013	0.034123203483389	0.003993203
− 2.4	0.03602	0.041024363064727	0.005004363
− 1.9	0.04622	0.051327181275063	0.005107181
− 1.4	0.06713	0.068122962680242	0.000992963
0	0.0006373	4.405924091149890E−04	0.000196708
0.05	0.05393	0.030903923489380	0.023026077
0.1	0.09238	0.060330336880640	0.032049663
0.2	0.1333	0.108035297115075	0.025264703
0.3	0.1431	0.133828195617252	0.009271804
0.4	0.1309	0.140001098562017	0.009101099
0.9	0.09977	0.098528514681519	0.001241485

**Table 2:** A comparison concerning RK-4 and averaging solutions ($a_{2}$ by $\sigma_{1}$).

$\sigma_{1}$	Numerical calculation	Averaging solution	Absolute error
− 5	0.0007756	0.00159446778063665	0.000819
− 4.5	0.001016	0.00196668462448009	0.000951
− 4	0.001367	0.00248630442207555	0.001119
− 3.5	0.001906	0.00324207062336632	0.001336
− 3	0.002758	0.00440060935300175	0.001643
0	0.2492	0.249236704179633	3.67E-05
0.5	0.09075	0.108361397040312	0.017611
1	0.03016	0.0363246714325892	0.006165
1.5	0.01336	0.0170592388013241	0.003699
2	0.007136	0.00977732901907154	0.002641
2.5	0.004275	0.00630920042671862	0.002034

Figure 16 shows the impacts of different values of $F$ on the FRC. With increasing amounts of $F$, the oscillation amplitude of the system increases, and the peaks of the curve grow up as shown in Fig. 16a.

For the amplitude of the control vibration, when the values of $F$ are improved, the peak of the curve grows up as illustrated in Fig. 16b on FRC curves earlier^15,24,25,37,38,41^.

Furthermore, in different earlier works^15,42^, the effect of $\gamma_{1}$ is shown in Fig. 17. From Fig. 17a, the two peaks of the curve grow up when the small values of $\gamma_{1}$ are selected. The bandwidth and flatness of the peak for the controller curve increase when the values of $\gamma_{1}$ are reduced, as indicated in Fig. 17b.

The effect of changing the values for $\gamma_{2}$ is indicated in Fig. 18. On decreasing the values of $\gamma_{2}$, the height of the two peaks of the system curve increases and the areas of instability appear as represented by Fig. 18a. The bandwidth for the peak of the controller curve increases around $\sigma_{1} = 0$ until it flattens and turns into two peaks on either side of that region, and the areas of instability appear at small values of $\gamma_{2}$ as shown in Fig. 18b. The previous mode is already provided^15^.

Figure 19 describes the effect of different values of $\lambda$ on FRC. From Fig. 19a, at small values of $\lambda$, the flatness and bandwidth of the two peaks for the curve of the structure shift to right. For Fig. 19b, it describes the flattening of the peak for the controller curve around $\sigma_{1} = 0$ until it becomes a peak to the right at a negative value of $\lambda$, while it turns to a peak to the left at a positive value of $\lambda$. Similar results were obtained earlier^42^.

From Fig. 20, there is no effect of $\delta$ on FRC.

Changing the values of $G_{1}$ and $G_{2}$ with their effect on FRC is demonstrated in Figs. 21 and 22, correspondingly. The bandwidth region of the system expands at large values of $G_{1}$ and $G_{2}$ as represented in Figs. 21a and 22a, respectively. The peak of the controller curve decreases and turns into two peaks on both sides of the bandwidth region with increasing the values of $G_{1}$, as illustrated in Fig. 21b. While the peak of the controller curve expands without height until it turns into two peaks with an increase in their height on both sides of the bandwidth region with increasing values of $G_{2}$, as shown in Fig. 22b. Similar illustrations were presented in the previous works^30^.

The effect of $\mu$ on FRC is shown in Fig. 23. With increasing the values of $\mu$, the two peaks of the system curve decrease as shown in Fig. 23a. From Fig. 23b, the peak of the controller curve is flattened until the two peaks appear on either side of the region $\sigma_{1} = 0$ at small values of $\mu$. Previous examples of the results were included^30^.

The effect of $\mu_{1}$ on FRC is represented in Fig. 24. By increasing the amounts of $\mu_{1}$, the two peaks of the structure curve go down and a jump phenomenon appears at the region $\sigma_{1} = 0$ as shown in Fig. 24a. From here, small values of $\mu_{1}$ should be chosen to a better reduction of the vibrations for the structure at the studied resonance situation. From Fig. 24b, the peak of the controller curve declines at large values of $\mu_{1}$. Earlier results in the frequency response curves were provided earlier^30^.

Figure 25 indicates the effect of different values of $\omega$ on FRC. From Fig. 25a, at small values of $\omega$, the height of the two peaks for the system curve increases. Simultaneously, the peak of the controller curve rises at small values of $\omega$ as represented in Fig. 25b as shown earlier^30^.

The effect of different values of $\sigma_{2}$ on FRC is exposed in Fig. 26. In this figure, it is realized that the entire curve moves when changing the values of $\sigma_{2}$. As the studied resonance case (i.e. $\sigma_{2} = \sigma_{1}$) is selected, the vibration of the system is suppressed as illustrated in Fig. 26a. Also, from Fig. 26b, when the studied resonance case (i.e. $\sigma_{2} = \sigma_{1}$) is selected, the peak of the controller curve moves in this way. Similar results were obtained earlier^15,30^.

**Figure 27:**
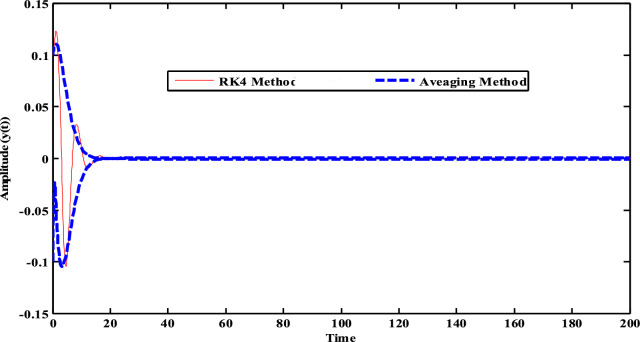
Time history comparison between RK-4 and an Averaging method at $\sigma = \omega$ and $\omega_{1} = \omega$.

Figure 27 shows a contrast concerning the perturbation procedures, as specified in Eqs. (36), (38), (40) and (41), and the calculation simulation as described in Eqs. (22) and (23) of the time history was achieved. The blue dashed lines display the modulation of the amplitude of the widespread coordinate. Furthermore, the red solid lines denote the time history of oscillations which display computationally the solutions of the structure with the NDF-control. Here is a better arrangement concerning the theoretical and computational solutions, which validates the approval of our solution.

**Figure 28:**
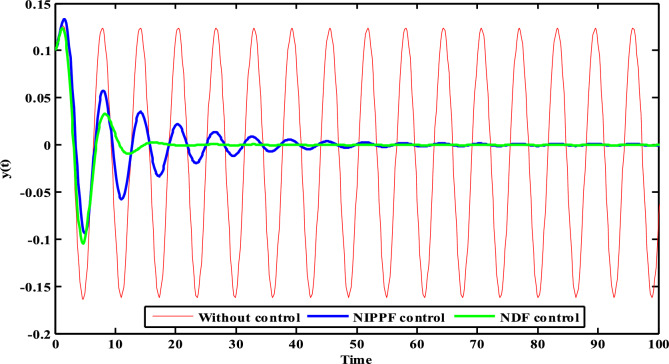
Time history contrast concerning various controls.

Figure 28 presents the contrast concerning diverse controls to confirm the presentation of the oscillation lessening that seems in the HRVD system deprived of a control and designated with the red line. The contrast is completed through NIPPF control with a blue line, and NDF with a green line.

## Conclusions

In the current work, the oscillating HRVD system with cubic-quintic nonlinear terms is examined theoretically and computationally, deprived of controller with an equivalent linear differential equation. The innovative approach also referred to as the “new methodology” or NPA just transforms the nonlinear ODE into a linear one. A new corresponding frequency that is similar to the linear ODE is produced. A thorough explanation of the NPA is included for the benefit to the readers. Using a numerical comparison performed by the MS, the theoretical results are confirmed. The precise numerical and theoretical solutions are both displayed outstanding consistency. When the restoring forces are present, as is commonly known, all classical perturbation approaches use Taylor expansion to augment these forces and, as a result, reduce the difficulty of the given problem. This shortcut is no longer available under the NPA. With accumulation, it is possible to properly evaluate the stability analysis of the NPA, which was not feasible with earlier conventional approaches. Therefore, the NPA is a more reliable source for examining approximations of solutions for strongly nonlinear oscillators. Due to its adaptability to novel nonlinear situations, the NPA is a useful tool in the fields of applied science and engineering. It should be noted that there some recent works have been published during this novel methodology^49–52^. Several plots curves are depicted to grantee the stability of the considered solutions. Since the unstable solutions are physically not preferred, no polar plots are graphed for the unstable case. The more details are emphasized with reference to the adopted distinctive technique. The approximation solution after the NDF control is achieved by adding an adapted averaging method. A computational approach based on numerical calculations is working to validate the preceding approximate solution. Additionally, each of the phase portraits and the linearized stability are planned. By contrast, the oscillation lessening for HRVD with NDF controller is proposed at the instantaneous main and 1:1 internal resonance. Furthermore, a group of sketches is completed to validate the FRC and various parameters by the MATLAB Software. The most significant findings of the study can be summarized in the following points:

1. The NDF controller contributed to dropping oscillations for the careful HRVD with a condensed proportion of 99.48%.
2. The amplitude of the HRVD is amplified as excitation force $F$ increases.
3. The cumulative amount of the factors $\gamma_{1} ,\gamma_{2} ,\mu ,\mu_{1} ,\omega_{1} \,\,{\text{and}}\,\,\omega$ yields a reduction in the amplitude of the HRVD.
4. The HRVD structure with a NDF control is grasped to the minimum amounts on the frequency response curve at $\sigma_{1} = \,\sigma_{2}$.
5. The bandwidth region progressively increases in the amplitude of the HRVD structure as the control factors $G_{1} \,{\text{and}}\,\,G_{2}$ rise.
6. Aimed at justification response curves, there are excessive arrangements concerning the estimations of FRC and RK-4 solutions as obtained in Fig. 14.
7. A Comparison between NIPPF and NDF controllers is presented to verify that the NDF control is the greatest controller approach that can be used to decrease the oscillations in the HRVD system as presented in Fig. 27.

### Supplementary Information

Supplementary Information.

## Data Availability

All data generated or analyzed during this study are included in this manuscript.
