# Supplementary material for: Different controllers for suppressing oscillations of a hybrid oscillator via non-perturbative analysis
Source: Sci Rep. 2024 Jan 3;14:307. doi: 10.1038/s41598-023-50750-9 (PMC10764832; doi:10.1038/s41598-023-50750-9)
Supplement: Supplementary file 1 — Supplementary Information. [file 41598_2023_50750_MOESM1_ESM.docx]

**Different Controllers for Suppressing Oscillations of a Hybrid Oscillator: Non-Perturbative Analysis**

Galal M. Moatimid, A. T. El-Sayed, and Hala F. Salman
Department of Mathematics, Faculty of Education, Ain Shams University, Cairo, Egypt

Department of Basic Science, Modern Academy for Engineering and Technology, Egypt

Department of Basic Sciences, Faculty of Computers and Artificial Intelligence, Cairo University, Giza, Egypt

E-mails

: gal_moa@edu.asu.edu.eg

Corresponding author: [ashraftaha211@yahoo.com](mailto:ashraftaha211@yahoo.com)

: halasalman35@yahoo.com

**Appendix**

The elements appearing in Eq. (49) may be listed as follows:

$r_{11}=-\mu-\frac{3\gamma_{1}a_{10}^{2}}{8}-\frac{9\gamma_{2}\omega^{2}a_{10}^{2}}{8}$, $r_{12}=\frac{F}{2\omega}\cos\theta_{10}$, $r_{13}=\frac{G_{1}\omega_{1}}{2\omega}\cos\theta_{20}$, $r_{14}=-\frac{G_{1}\omega_{1}}{2\omega}a_{20}\sin\theta_{20}$,

$r_{21}=\frac{\sigma_{1}}{a_{10}}-\frac{9\lambda a_{10}}{8\omega}-\frac{25\delta a_{10}^{3}}{16\omega}$, $r_{22}=-\frac{F}{2\omega a_{10}}\sin\theta_{10}$, $r_{23}=-\frac{G_{1}\omega_{1}}{2\omega a_{10}}\sin\theta_{20}$, $r_{24}=-\frac{G_{1}\omega_{1}}{2\omega a_{10}}a_{20}\cos\theta_{20}$,

$r_{31}=-\frac{G_{2}\omega}{2\omega_{1}}\cos\theta_{20}$, $r_{32}=0$,$r_{33}=-\mu_{1}$, $r_{34}=\frac{G_{2}\omega}{2\omega_{1}}a_{10}\sin\theta_{20}$,

$r_{41}=\frac{\sigma_{1}}{a_{10}}-\frac{9\lambda a_{10}}{8\omega}-\frac{25\delta a_{10}^{3}}{16\omega}+\frac{G_{2}\omega}{2\omega_{1}a_{20}}\sin\theta_{20}$, $r_{42}=r_{22}$, $r_{43}=-\frac{G_{1}\omega_{1}}{2\omega a_{10}}\sin\theta_{20}+\frac{(\sigma_{2}-\sigma_{1})}{a_{20}}$,

$r_{44}=-\frac{G_{1}\omega_{1}}{2\omega a_{10}}a_{20}\cos\theta_{20}+\frac{G_{2}\omega}{2\omega_{1}a_{20}}a_{10}\cos\theta_{20}$.
